# Increased Cost of Motor Activity and Heat Transfer between Non-Shivering Thermogenesis, Motor Activity, and Thermic Effect of Feeding in Mice Housed at Room Temperature – Implications in Pre-Clinical Studies

**DOI:** 10.3389/fnut.2016.00043

**Published:** 2016-10-06

**Authors:** Patrick C. Even, Anne Blais

**Affiliations:** ^1^UMR Physiologie de la Nutrition et du Comportement Alimentaire, AgroParisTech, INRA, Université Paris-Saclay, Paris, France

**Keywords:** mouse, indirect calorimetry, spontaneous motor activity, cost of activity, thermal regulation

## Abstract

The components of energy expenditure, total metabolic rate (TMR), resting metabolic rate (RMR), thermogenic response to feeding (TEF), activity, and cost of activity were measured in fed and fasted mice housed at 22 and 30°C. Mice housed at 22°C had more than two times larger TMR and RMR. Mice at 22°C were less active when fasted but more active when fed. Cost of activity was nearly doubled in the fasted and in the fed state. Analysis of the short-term relation between TMR, RMR, and bouts of activity showed that, at 22°C, the bouts of activity induced a decrease in the intensity of RMR that reflected the reduced need for thermal regulation induced by the heat released from muscular contraction. This phenomenon induced a considerable underestimation of TEF and prevented its reliable measurement when mice were housed at 22°C. Correlation between TMR and activity measured across time in individual mice was very strong at both 22 and 30°C, but the correlation measured across mice was much weaker at 30°C and no longer significant at 22°C. We suspect that this phenomenon was due to the fact that RMR is a much more reliable predictor of TMR than activity. RMR is more variable at 22°C than at 30°C because of heat transfers between thermal regulation and heat released by other discontinuous processes, such as activity and TEF. Therefore, more noise is introduced into the correlations performed across multiple mice between TMR and activity at 22°C. On the other hand, it should be kept in mind that the doubling of TMR and RMR at 22°C is fueled by an increased non-shivering thermogenesis that can obviously modify how the mouse responds to pharmacological and nutritional challenges. Taken together, these results suggest that in pre-clinical studies, mice should be housed in conditions where thermal regulation is limited as is generally the case in humans. However, the increased sensitivity of mice to small changes in ambient temperature can also be used as a versatile tool to investigate the role of thermal regulation on the energy balance equation in humans.

## Introduction

Obesity research to get a mechanistic understanding and to provide guidelines for clinical investigations has used mainly mouse models for experiments that are not ethical in humans. No other animal model offers such large possibilities of phenotyping in response to metabolic, genetic, and behavioral manipulations (1). However, it is important that mouse and human biology are similar in order to get reliable predictive values from mouse experiments.

Energy balance is determined by the equilibrium between energy intake and energy expenditure from basal metabolic rate, thermic effect of feeding, cost of activity, and thermoregulation. In obesity research, it appears that the cost of thermoregulation was until recently an underestimated component despite the fact that it is widely acknowledged that in small endothermic rodents energy demand to maintain body temperature can become an important component of the energy Budget (2). In contrast, humans who have a mass about 3000-fold larger than mice live predominantly close to thermo-neutrality. Humans maintain their body temperature without thermoregulatory effort, the heat generated by ongoing metabolism is the only process needed. The mouse is very sensitive to ambient temperatures that decrease below thermal neutrality (28–31°C) because of its small size, and therefore very large body surface area to mass ratio. To maintain their body temperature, mice rely heavily on thermogenic processes specifically devoted to heat production, which are mainly uncoupled respiration in brown adipose tissue (3, 4), but also depend on shivering thermogenesis and heat generated by muscular activity (5, 6).

In most cases, mice studies have been conducted at temperatures of 20–22°C, which is far below their thermal neutrality (30–32°C). This condition increases the cost of thermoregulation that can double energy requirements (7) and subsequently increases food intake, sympathetic activity, blood pressure, and heart rate. Therefore, the question is raised whether this large amount of energy produced to maintain body temperature can affect not only resting metabolism but also the amount and the cost of locomotor activity, the thermogenic response to feeding (TEF), and more generally the responses to various metabolic challenges. Indeed, the extra heat produced by the activity cost (physical work is only ~20% efficient) and TEF (enzymatic reactions are ~60% efficient) can potentially reduce the energy required for thermoregulation. Therefore, it is possible to consider that heat released by activity or feeding will reduce the cost of thermoregulation and can induce an underestimation of activity or feeding costs. This phenomenon was suggested in a previous paper which showed that TEE was correlated with activity when mice were housed at 30°C but not when they were housed at 20°C. At this lower temperature, energy expended from activity was masked by the reduction of the energy expended for thermoregulation (8). Moreover, evaluation of drug effects on energy expenditure may be altered when mice are housed at room temperature because the compensatory reduction in cold-induced thermogenesis can offset the drug-induced increase. It has been suggested also that when mice are housed below thermal neutrality, BAT thermogenesis may play an important role in food intake control and energy balance regulation (9). An inadequate response to cold was reported also in Lep^ob^/Lep^ob^ mice (10), which may explain why it is only at temperatures below thermal neutrality that they have a lower energy expenditure than wild-type mice (11, 12).

According to these results, analysis of the preliminary results of a current study lets us suspect that ambient temperature could have profound effects on the mechanisms of adaptation of mice to low-protein diets. We extracted the control mice of this study to focus on the evolution of energy metabolism components when mice are acclimatized to the vivarium temperature (22°C) or at thermal neutrality (30°C). In this article, we report changes induced on resting and total metabolic rate (TMR), spontaneous motor activity, cost of activity, and the TEF.

## Materials and Methods

### Animals and Housing

Twenty-one female Balb/cOlaHsd mice were singly housed in a conventional facility with a reversed 12:12-h dark–light cycle (lights on at 20:00 hours). All experimental procedures complied with institutional guidelines and policies to prevent pain and distress under license from the French Veterinary Service (Ethics committee agreement number 12-095 and 13-012). The mice were provided by Harlan Laboratories (France) at 7 week of age and were allowed 2 weeks adaptation to the laboratory conditions before any experimental manipulation.

The mice used in this study were the control mice of two different experiments performed in 2014 and 2016. Water and food were provided *ad libitum* during the two studies unless otherwise stated. Mice were fed either a soy protein or a casein diet [by energy: soy protein or casein 24%, carbohydrate 66% (56.4% corn starch, 9.6% sucrose), fat (soy oil) 10%]. Food quotient of the two diets was 0.93. The results of the casein and soy-protein fed mice were pooled after we controlled for the similar reactivity of the two groups to the differences in ambient temperature.

**Figure 1:**
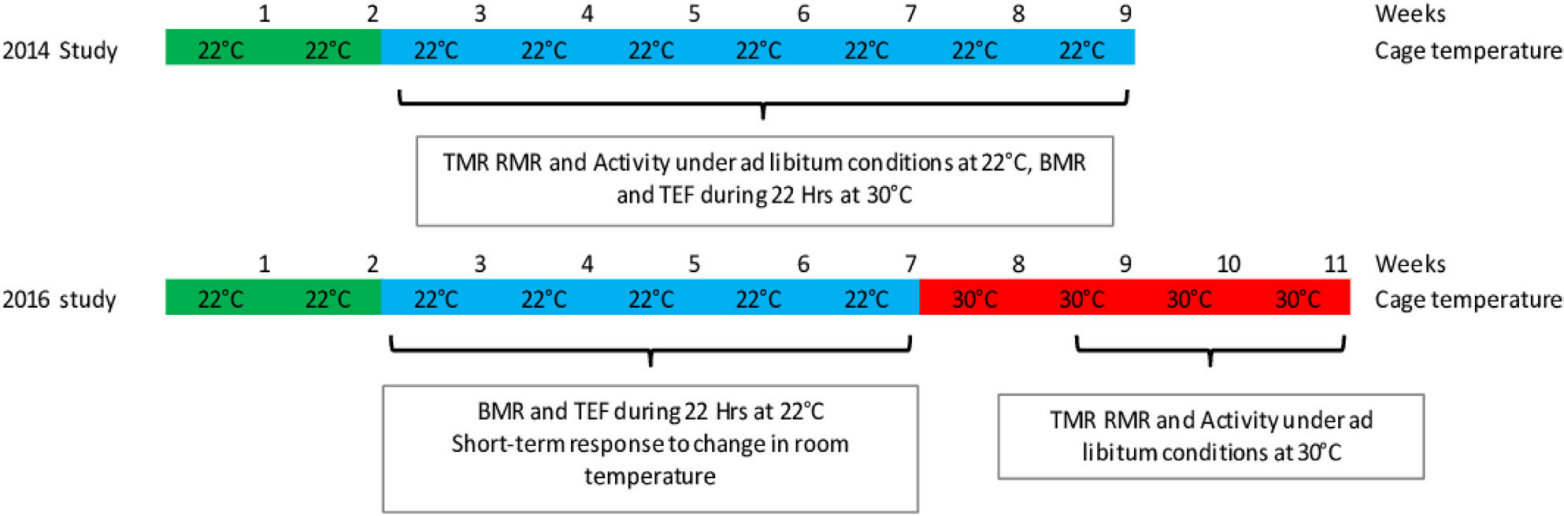
**Experimental design of the two studies**.

In the first study (*n* = 11, 6 soy and 5 casein), the mice were housed continuously at 22°C. In the second study (*n* = 10, 5 soy and 5 casein), the mice were first housed at 22°C during 5 weeks, then the room temperature was increased to 30°C, and the mice maintained under these housing conditions for four more weeks (Figure 1).

### Indirect Calorimetry

The indirect calorimetry system used in this study was a custom designed system working in pull mode and described in detail in several previous publications (13–15). Respiratory quotient (RQ) was calculated as the ratio of CO_2_ production (VCO_2_) over O_2_ consumption (VO_2_). Metabolic rate was calculated in watts (W) using the Weir equation (14). Spontaneous activity was measured by force transducers located under the floor of the cage. Data acquisition and data processing were performed by computer programs developed in the laboratory and written in the LabVIEW^®^.

In a first study, TMR and spontaneous activity (Act) were measured in *ad libitum*-fed mice housed at 22°C, and the TEF was measured at 30°C. In a second study, mice followed the reverse procedure, i.e., TEF was measured in mice housed at 22°C, and TMR and activity were measured under *ad libitum* conditions at 30°C after the mice were accustomed for at least 10 days to this temperature (Figure 1).

### Measurement of TMR, RMR, and Activity under *Ad Libitum* Conditions

Mice were previously accustomed for 3–4 days to the calorimetry procedure by being housed in the same cages as used for the calorimetry recording. For the recording, they were kept in the same cage, and the cages were connected to the calorimetry system.

VO_2_, VCO_2_, and intensity of spontaneous motor activity were recorded from five chambers (2.5 L volume, constant flow rate of 600 mL/min). Each chamber, then room air (to correct for background VO_2_–VCO_2_) was sampled during 100 s, so that each cage was sampled every 10 min. In the cages, a sheet of blotting paper was used as bedding. Water and food were freely available in small boxes fixed on the side of the chambers. Data acquisition was performed without interruption during 2 days. Day 2 was used for data analysis.

**Figure 2:**
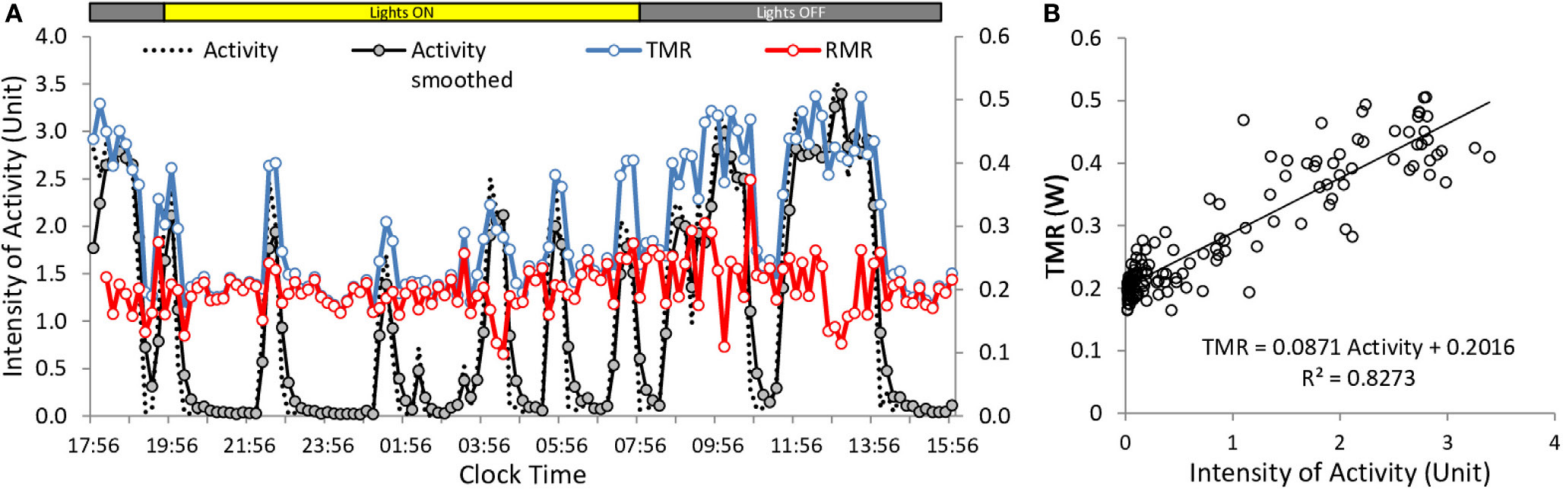
**Example of calculation of activity cost and RMR in mice under *ad libitum* conditions**. Data were obtained from the multiplexed device. Acquisition was at 10 min intervals. **(A)** Changes in activity, TMR and RMR measured along the time. The original activity trace was slightly smoothed to better correlate with TMR. **(B)** Correlation between intensity of activity and TMR. Slope of the regression gives the cost of activity and origin gives RMR. RMR in **(A)** was computed as TMR (activity × cost).

Analysis of the components of energy expenditure provided TMR and resting metabolic rate (RMR), RQ, and intensity of activity. The relation between TMR and activity was computed across time for each mouse. To improve the correlation between changes in the intensity of TMR and changes in the intensity of activity, a slight convolution of the activity signal was performed in order to reproduce the smoothing of the respiratory response induced by the dead space of the chambers (Figure 2A). RMR was obtained as the *Y*-axis intercept of the correlation between TMR and activity, and the cost of activity was computed as the slope of the correlation between TMR and activity (Figure 2B). The metabolic rate of activity (AMR) was computed as the difference between TMR and RMR.

The relations between TMR, AMR, and activity and TMR and RMR were also computed across multiple mice by using mean daily TMR, AMR, RMR, and activity values obtained in each mouse.

During experiment 1, temperature in the experimental room was regulated at 20°C in order to maintain a temperature of 21–22°C in the metabolic chambers. During experiment 2, mice were previously acclimatized for at least 1 week at a room temperature of 30°C, and temperature in the experimental room was maintained at 29°C in order to maintain a temperature of 30–31°C in the metabolic cages.

### Measurement of the Thermic Response to Feeding and of the Short-Term Changes Induced by Activity on TMR, RMR, and RQ during a Cycle of Fasting and Refeeding

**Figure 3:**
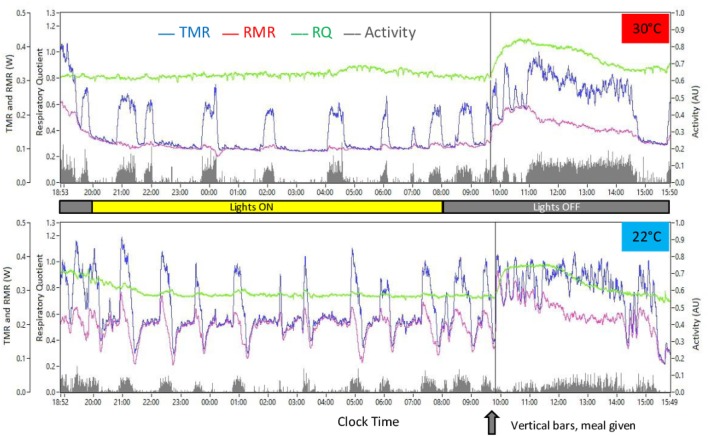
**Example of results observed in response to ingestion of a meal-test after overnight (mainly during the light period for the mouse) food restriction (screen copies of the computer program developed in the laboratory to edit the calorimetry data)**. Top, mouse housed at 30°C (2014 study); bottom, mouse housed at 22°C (2016 study). VO_2_, VCO_2_, and activity data were initially recorded at 2 s intervals then processed by the method of Kalman to compute TMR and RMR. RQ was computed as VCO_2_/VO_2_. Vertical bars crossing the figures indicate time when the meal was introduced into the cage.

These measurements were performed by measuring VO_2_, VCO_2_, and spontaneous motor activity continuously on one single cage at 2 s intervals. The uninterrupted acquisition on one cage and the high frequency of data sampling were required to perform a detailed analysis of the short-term relation between changes in VO_2_ and VCO_2_ and intensity of activity in order to be able to precisely compute the energy cost of activity and subsequently to compute RMR and TEF without artifacts due to variability in spontaneous activity. This process is based on a filtering procedure according to the method of Kalman and has been described in detail in several previous publications from our laboratory (14, 15) and more recently in one by Van Klinken and colleagues (16). Examples of results on individual mice are given Figure 3. Temperature in the cage was adjusted by decreasing room temperature below the required value in the cage and heating the wall of the cage with a heating coil controlled by a temperature gage. This system allowed the temperature (±0.2°C) to be maintained stable in the cage.

Mice were housed in the cage between 17:00 and 18:00 hours with water but no food and were kept overnight (i.e., mostly during their light period) in the metabolic cage (Figure 3). The next morning, a calibrated meal of 1 g (16 kJ) was introduced into the cage without interrupting data acquisition, and data recording was continued during 6–7 h. Average RMR and RQ during the 2 h that preceded the meal were used as baseline RMR and RQ values to calculate the changes induced by ingestion of the meal. TEF was computed in kJ as the cumulative increase above baseline RMR during 6 h after meal delivery. Short-term changes in TMR, RMR, and RQ in relation to the bouts of activity were studied by pooling activity periods extending from one hour before to one after well differentiated bouts of spontaneous activity that occurred between 23:00 and 08:00 hours, i.e., in fasted mice in the post-absorptive state and after non-shivering thermogenesis (NST) had time to switch off in mice housed at 30°C (see Figure 3). Hourly changes in the intensity of activity were also computed to compare intensity of spontaneous activity during fasting and refeeding.

All experiments were performed in mice usually housed at 22°C. During experiment 1, temperature in the metabolic cage was regulated at 30°C; during experiment 2, temperature was regulated at 22°C.

### Statistical Analysis

Data are presented as mean ± SEM. Values at 22 and 30°C were compared using a Student’s *t*-test in Excel, or by two-way ANOVA in R^®^ when means were compared in relation to a third parameter (time for TEF, distribution classes for TMR activity, and RQ). Significant ANOVA results were followed using *post hoc* Tukey tests. Significance was set at *P* < 0.05.

## Results

### RMR, RQ, Activity, and TEF in Fasted-Refed Mice

**Figure 4:**
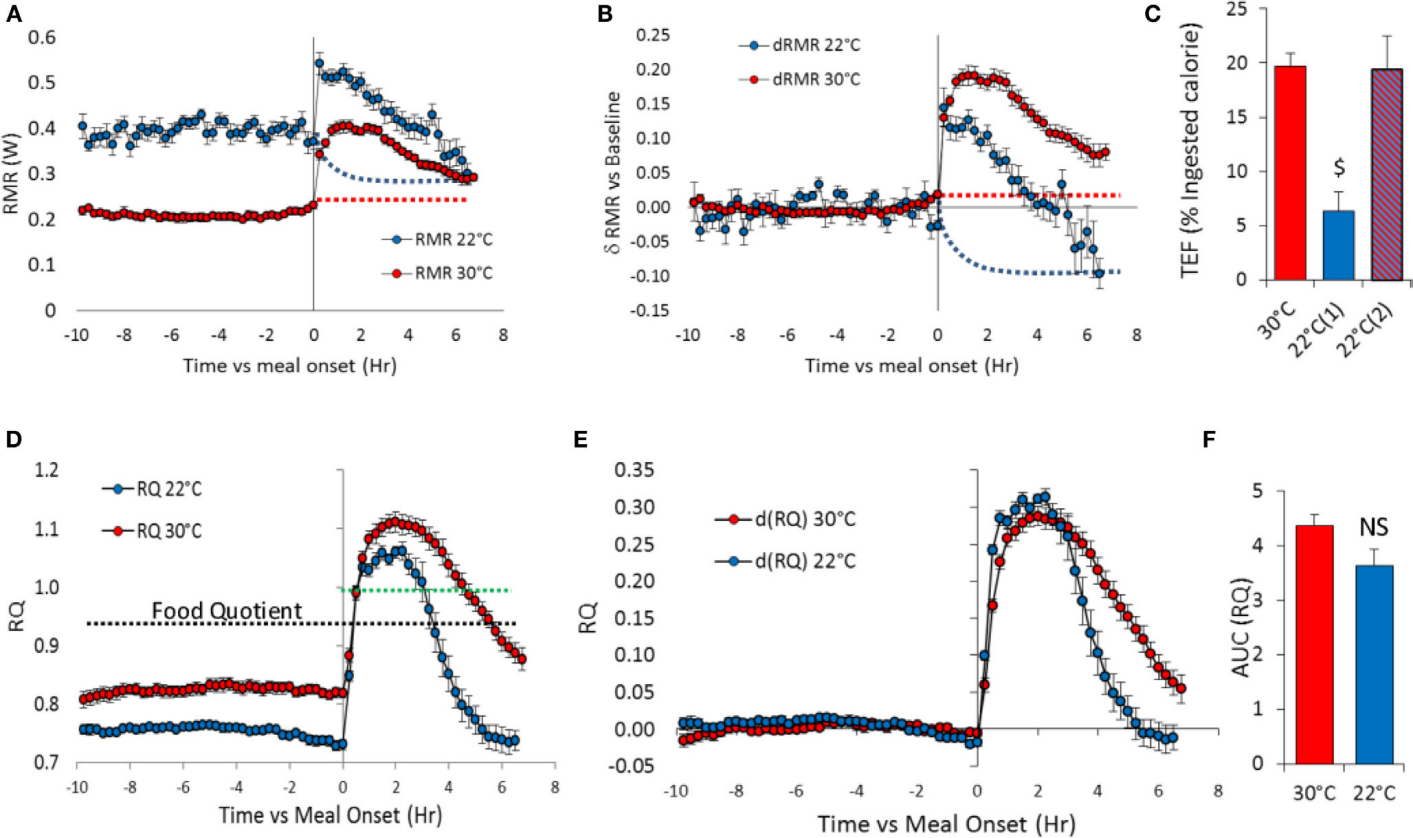
**Meal induced changes in TMR, RMR (watts), and RQ in overnight fasted mice at 30°C (*n* = 11) and 22°C (*n* = 10)**. Test-meal (1 g, 16 kJ) given at *t* = 0. **(A)** Absolute TMR and RMR values, **(B)** TMR and RMR values relative to pre-meal values, **(C)** TEF computed as the cumulative increase in RMR over pre-meal values [30 and 22°C(1)] or after taking into account the possible decrease in RMR at 22°C [22°C(2)] [$: *P* < 10^−5^ vs. 30 and 22°C(2)]. See TEF in kJ Table 1. Red dashed lines – extrapolation of fasting RMR at 30°C. Blue dashed lines – estimated decrease in RMR induced by activity and TEF used to adjust the calculation of TEF at 22°C [22°C(2)] **(D)** Absolute RQ values. Black dashed lines – food quotient (0.93). Green dashed lines – figures the RQ value of 1. **(E)** RQ values above 1 imply that part of the ingested carbohydrates is converted to lipids (lipogenesis). RQ changes relative to pre-meal RQ values, **(F)** Area under curve (AUC) computed as the cumulative changes in RQ over pre-meal values.

**Figure 5:**
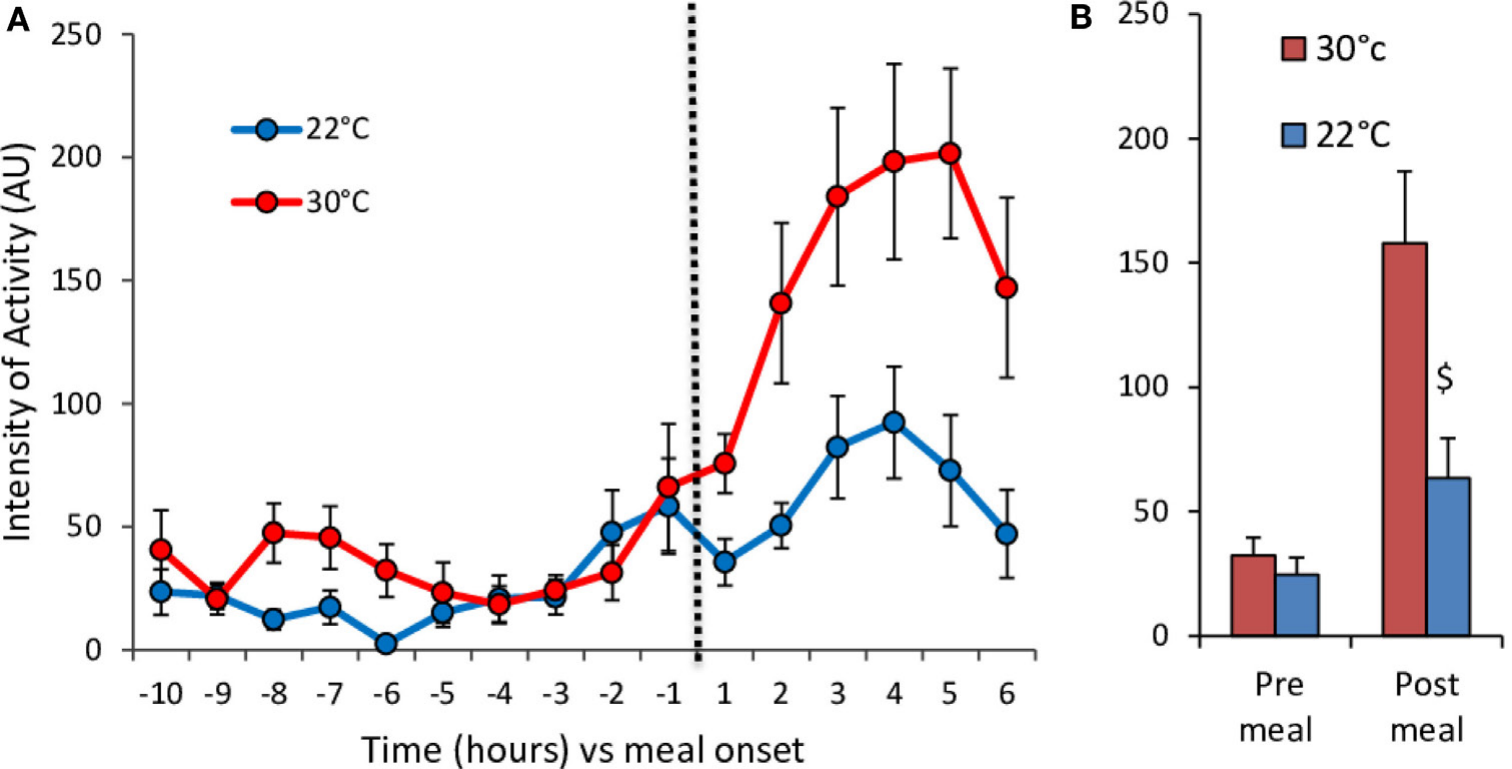
**Hourly changes in the level of spontaneous activity (A) and mean activity values (B) before (pre-meal) and after (post-meal) delivery of the test-meal at 30°C (*n* = 11) and 22°C (*n* = 10)**. **(A)** Time effect, *P* < 10^−15^; temperature effect, *P* < 10^−9^; time × temperature effect, *P* < 10^−4^. **(B)** $, *P* < 0.01.

Analysis of the changes in RMR and RQ during a cycle of fasting and refeeding showed large differences between mice housed at 22°C compared to those at 30°C. RMR measured after an overnight fast during the last 3 h before refeeding (~06:30–09:30) was nearly twice as large in mice housed at 22°C (Table 1; Figure 4A). Conversely, RQ was significantly lower attesting a greater reliance on fat derived substrates (Table 1; Figure 4D). During the fasting period (~23:00–10:00), activity was low and not significantly different at 30 and 22°C (Figures 3 and 5).

**Table 1:** **Components of energy expenditure in fasted-refed mice**.

	30°C (*n* = 11)		22°C (*n* = 10)				
	Mean ± SEM		Mean ± SEM		°C	Time	°C × T
Fasting RMR (W)	0.213	0.006	0.397	0.011	<10^−9^	–	–
Fasting TMR (W)	0.273	0.015	0.492	0.015	<10^−12^	–	–
TEF (kJ)	3.149	0.196	1.009	0.295	<10^−15^	<10^−15^	<10^−12^
TEF (% ingested)	19.68	1.22	6.309	1.845	<10^−15^	<10^−15^	<10^−12^
RQ	0.823	0.008	0.748	0.005	<10^−6^	–	–
AUC RQ	4.374	0.205	3.644	0.276	<10^−12^	<10^−2^	0.99

*TMR and RMR values are adjusted to 20 g BW. AUC, area under curve*.

Meal-induced increase in RMR was greatly reduced in mice housed at 22°C (Table 1; Figure 4B), and therefore, TEF computed by extrapolating pre-meal RMR appeared three times smaller [Table 1; Figure 4C; 22°C(1)]. However, it appeared that post-meal RMR was lower than pre-meal RMR 4 h after the meal and onward, suggesting that during the post-prandial period, the extra heat released by activity and TEF decreased the extra-energy expended for thermal regulation. Therefore, computing TEF by extrapolating pre-meal RMR probably underestimated TEF at 22°C. If RMR measured 6 h after meal onset was used as baseline, then TEF at 22°C was similar to TEF at 30°C [Figure 4C, 22°C(2)].

The meal-induced increase in RQ was of similar amplitude at 22 and 30°C but was of significantly shorter duration in mice housed at 22°C (Table 1; Figures 4E,F). After ingestion of the test-meal, and until the end of the experiment, spontaneous activity was significantly higher in mice housed at 30°C (Figures 3 and 5).

### Relation between TMR, RMR, RQ, and Activity Measured across Time in Fasted and Fed Mice

#### Fasted Mice

**Figure 6:**
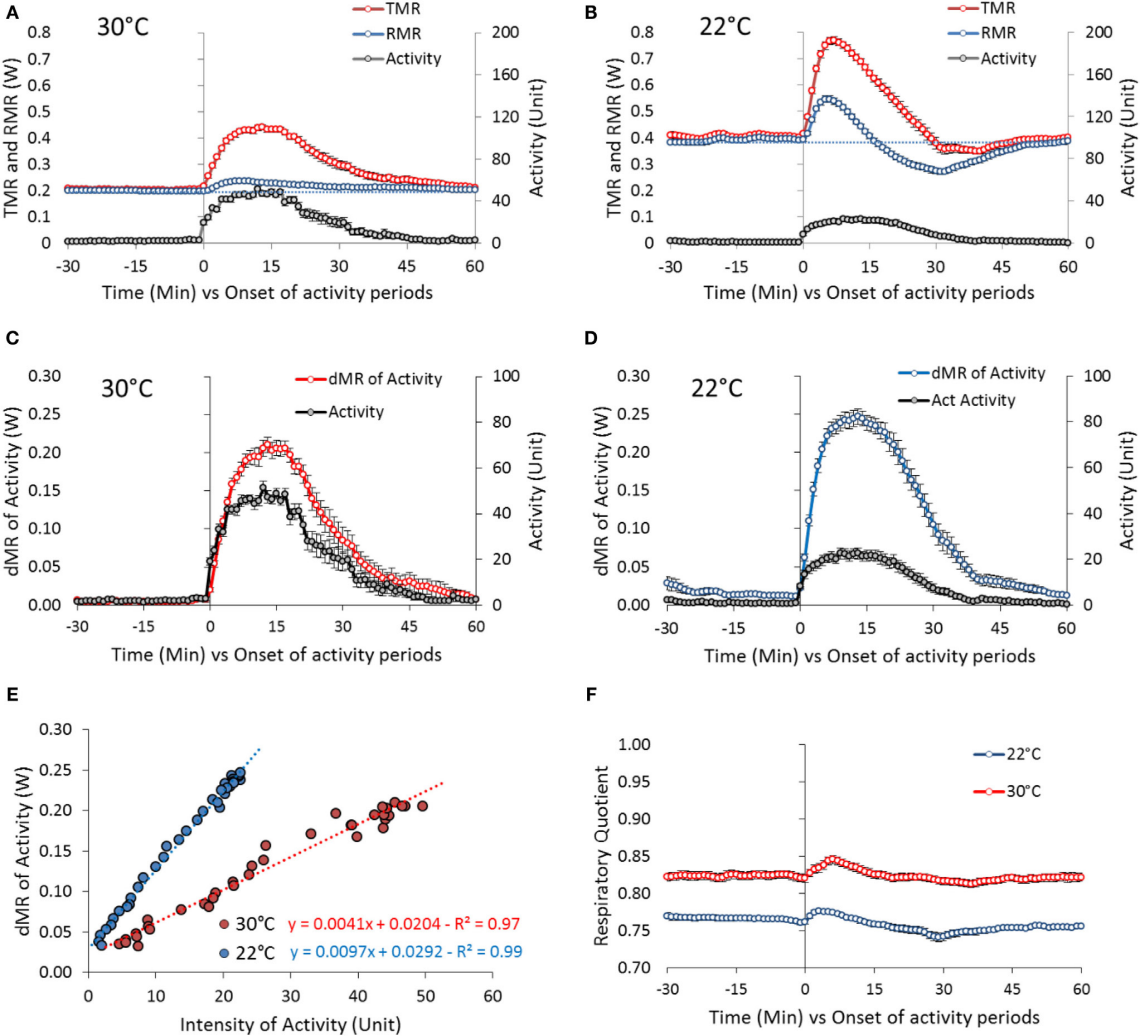
**Activity-induced changes in TMR and RMR in mice housed at 30°C (31 periods of activity from 11 mice) and 22°C (42 periods of activity from 10 mice)**. The periods of activity have been chosen as periods of well differentiated bouts of activity, preceded and followed by at least 1 h of quite complete rest that occurred during the overnight fast and the morning before the test-meal. The first 5 h of recording after the mice were housed in the metabolic chamber (18:00–23:00 hours) were discarded to focus on the response of mice in the post-absorptive state and adapted to the temperature in the cage (see Figure 3). **(A,B)** Absolute changes in TMR and RMR at 30 and 22°C. Blue dashed line: extrapolation of pre-activity RMR values. **(C,D)** Changes in δMR (δMR = TMR − RMR and reflects the true direct activity cost). **(E)** Correlation between activity and δMR changes at 30 and 22°C. **(F)** Absolute changes in RQ.

In mice housed at 30°C, the mean peak intensity of the bouts of activity was ~40 U and occurred 15 min after the onset of activity (Figure 6A). Mean duration of the activity periods was 30–40 min. The bouts of activity-induced parallel changes in the intensity of TMR, reflecting the metabolic cost of activity but only marginally modified the intensity of RMR. A very small but significant increase was however observed during the first min of activity [0.0347 ± 0.0037W, *P* < 10^−5^ (+17% vs. baseline)].

At 22°C, the mean peak intensity of the bouts of activity was very significantly reduced down to one half of the intensity observed at 30°C (~20 U) (Figure 6B). Activity increased TMR but also profoundly affected the evolution of RMR: RMR increased very significantly during the first 5 min of activity [0.146 ± 0.013 W, *P* < 10^−16^ (+ 37% vs. baseline)] then decreased progressively down to a value lower than before the onset of activity. The decrease lasted as long as the activity duration. After activity stopped, RMR increased progressively again and returned to pre-activity values in 30 min.

As we observed that the increase in TMR appeared of similar amplitude at 22 and 30°C despite the very significant decrease in the intensity of the bouts of activity, we calculated more precisely the cost of activity by processing the differences between RMR and TMR (δMR) in relation to activity (Figures 6C,D). This data processing confirmed that the cost of activity was higher at 22°C than at 30°C. The correlation between δMR and activity computed during the first 15 min of activity where the correlation was the best indicated a doubling of the metabolic cost of activity (Figure 6E).

As already quoted, RQ was significantly lower in mice housed at 22°C (Figure 6F) indicative of a greater reliance of fat derived energy. Activity induced transient changes of small amplitude that were similar at 22 and 30°C indicating that muscle contraction was fueled by the available substrate mix as used by the other tissues of the body.

#### Fed Mice

In fed mice, despite the fact that data acquisition was performed at a lower frequency and that the mice were fed, the correlations measured across time in each mouse between TMR and activity remained high (0.85 < *R* < 0.87) (Table 2, Figure 2B). This allowed the recording of fairly precise and reproducible values for RMR and cost of activity from the origin and from the slope of the correlations, respectively. TMR, RMR, and daily activity were significantly higher in mice at 22°C, and the cost of activity was approximately two times larger at 22°C than at 30°C (Table 2) as already observed in fasted mice. The AMR computed as TMR minus RMR was also significantly increased.

**Table 2:** **Components of energy expenditure in *ad libitum* fed mice**.

	30°C (*n* = 9)		22°C (*n* = 11)		
	Mean ± SEM		Mean ± SEM		*P*
TMR (W)	0.244	0.024	0.686	0.020	<10^−9^
RMR (W)	0.167	0.018	0.510	0.016	<10^−10^
RMR (% TMR)	68.02	1.56	74.43	0.72	<10^−2^
Activity (AU)	0.945	0.095	1.463	0.102	<10^−2^
AMR (W)	0.077	0.007	0.175	0.006	<10^−8^
AMR (% TMR)	31.98	1.56	25.57	0.72	<10^−2^
Cost of act (W/AU)	0.083	0.008	0.123	0.007	<10^−3^
Cor. Coef. between TMR and activity	0.869	0.015	0.856	0.008	NS

*TMR and RMR are adjusted to 20 g BW. N = 9 instead of 10 at 30°C because recording of the activity signal failed on one cage*.

### Relation between TMR, RMR, AMR, and Activity Measured across Multiple Mice in *Ad Libitum* Fed Mice

**Figure 7:**
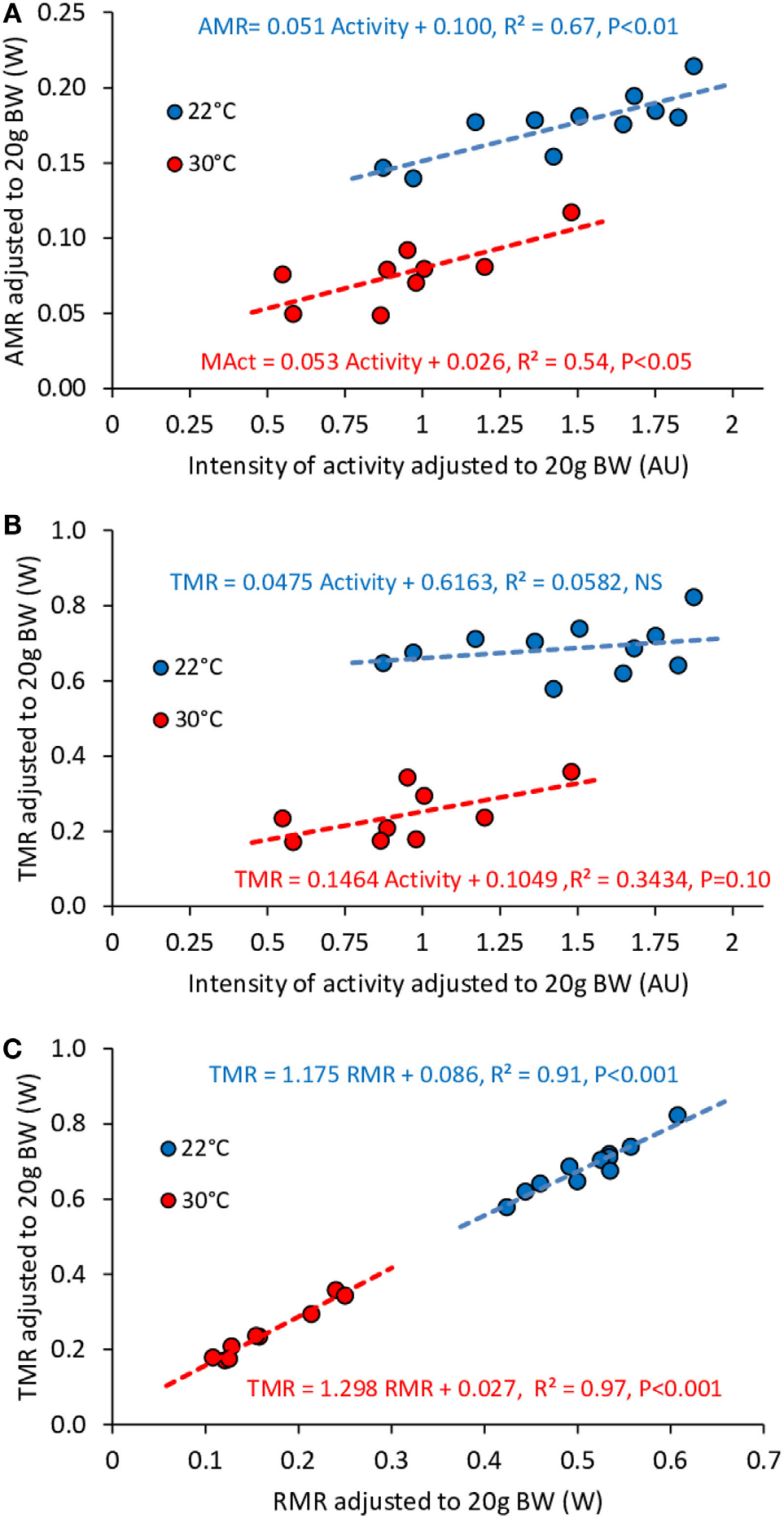
**Correlation between AMR and activity (A), TMR and activity (B), TMR and RMR (C) in *ad libitum* fed mice housed at 30°C (*n* = 9) and 22°C (*n* = 11)**. Data are adjusted to 20 g BW. Despite the fact that intensity of activity affects significantly AMR, the consequences on TMR are not significant. The strongest predictor of TMR appears to be RMR.

We observed a significant effect of activity on AMR at both 22 and 30°C (Figure 7A). However, activity did not significantly affect TMR at 22°C while the effect at 30°C was reduced and remained only borderline significant (Figure 7B). On the other hand, we observed a very strong correlation between TMR and RMR (Figure 7C), which may be related to the fact that RMR accounted for 68–74% of TMR (Table 2).

### Distribution of TMR and RMR Values in *Ad Libitum* Fed Mice

**Figure 8:**
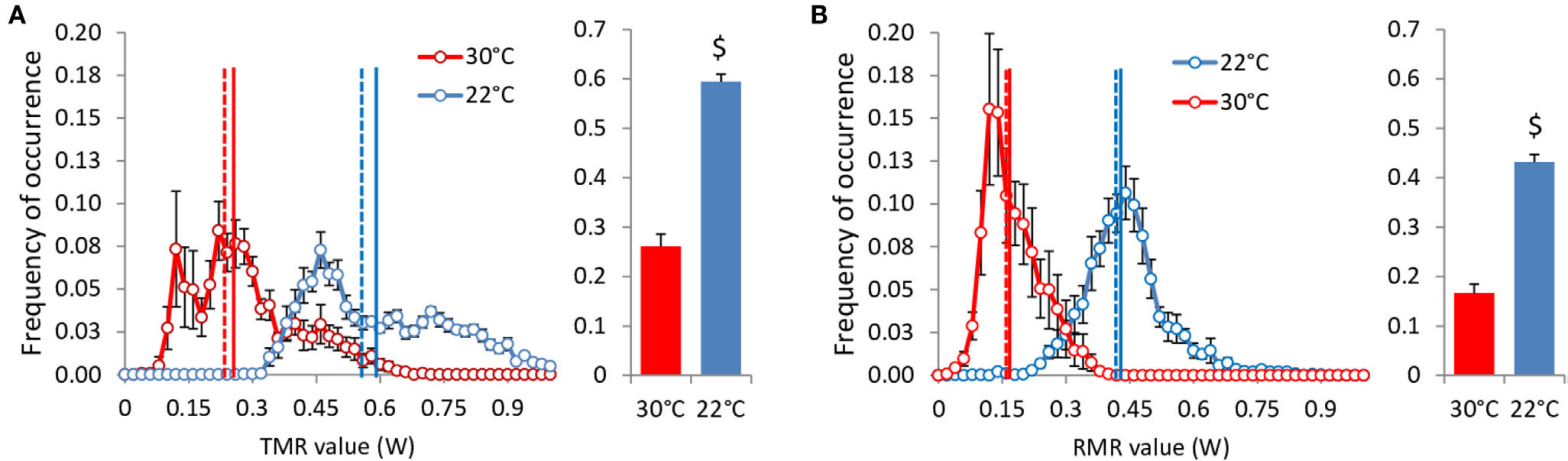
**TMR (A) and RMR (B) values distribution adjusted to 20 g BW in *ad libitum* fed mice housed at 30°C (*n* = 9) and 22°C (*n* = 11)**. Vertical lines: mean (solid) and median (dashed). $: *P* < 10^−5^.

Total metabolic rate and RMR values of *ad libitum* fed mice were more than doubled at 22°C (Table 2; Figure 8). This result is related to a strong shift to the right of the distribution frequency at 22°C. The distributions of TMR values are also less Gaussian than the distribution of RMR values, with a shift to the right that reflects the energy expended with activity, more pronounced in mice housed at 22°C. This shift induced a clear separation between mean and median activity values that was hardly visible on RMR. Unexpectedly, the distribution of RMR values at 30°C showed a peak at 0.15 W, i.e., at a value lower than RMR values measured after an overnight fast (Table 1), which suggests that at 30°C, under close to usual living conditions and despite continuous access to food, mice had possibly periods of very low metabolism.

### Distribution of Activity Values in *Ad Libitum* Fed Mice

**Figure 9:**
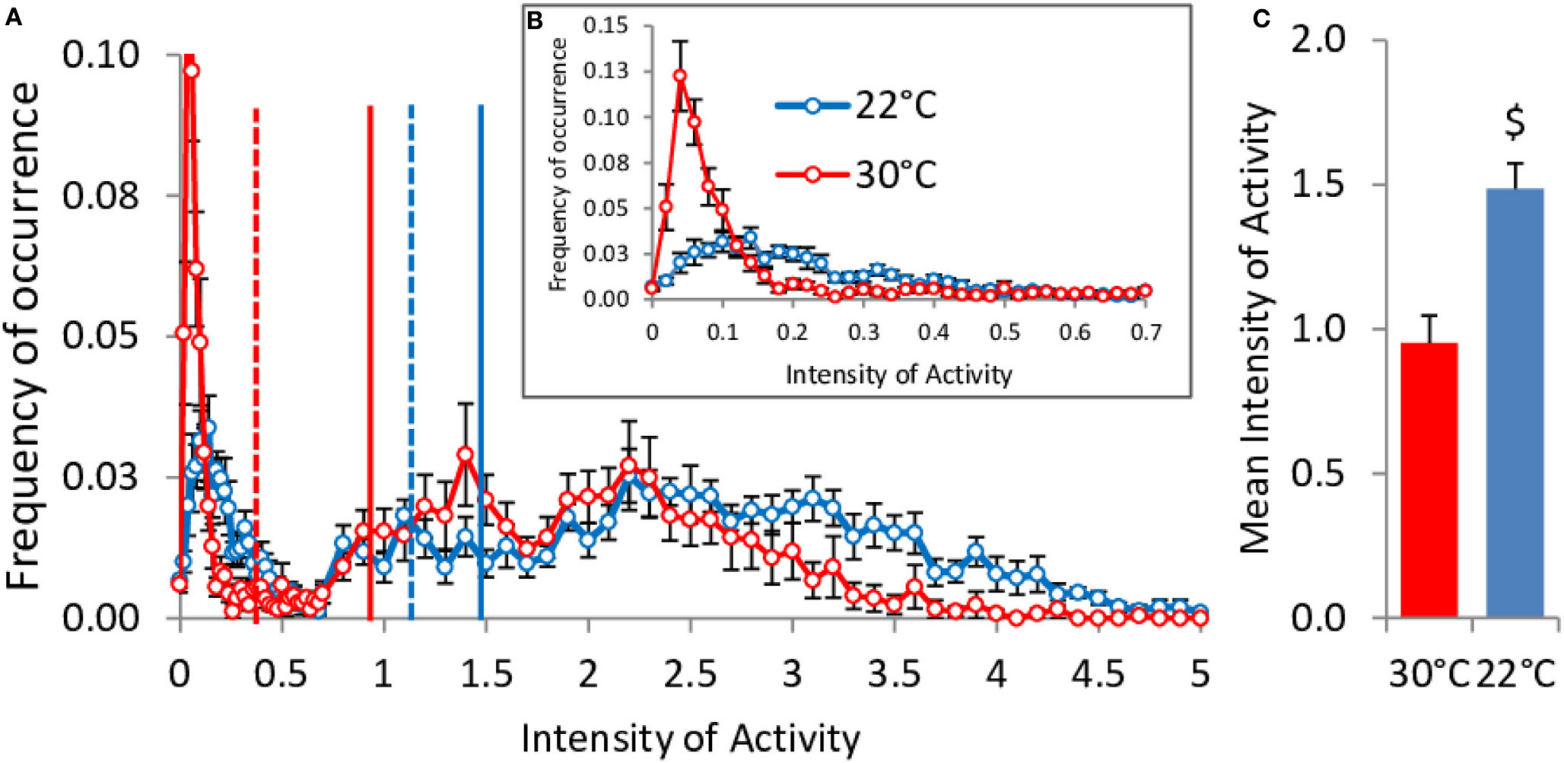
**Activity values distribution in *ad libitum* fed mice housed at 30°C (*n* = 9) and 22°C (*n* = 11)**. Vertical lines: mean (solid) and median (dashed). **(A)** Overall distribution of the activity values (*P* < 10^−6^ 22°C vs. 30°C). Inbox **(B)**, zoom of the distribution on the lower intensities of activity values. **(C)** Mean activity values ($, *P* < 0.001).

Contrary to what was observed in food restricted mice, spontaneous activity was larger at 22°C when mice were fed *ad libitum* (Figure 9C). This increase relies on both more activity of high intensity (above 3 U, Figure 9A) and on the fact that, at 22°C, the mice were never quite completely restless and therefore had a much smaller peak of low activity values than mice at 30°C. The strong shift to the left, down to 0.4 AU, of the median activity intensity when mice were housed at 30°C shows that they were completely inactive half of the time. Accordingly, Figure 9B shows that the occurrence of activities of very low intensities (between 0 and 0.1) amounted to 38% of the time in mice housed at 30°C, while it was only 12% in mice housed at 22°C. In contrast, mice at 22°C exhibited increased occurrence of activities of intensities between 0.1 and 0.4 (27% of time vs. 12%, Figure 9B) testifying to a form of restlessness.

## Discussion

This study confirms that energy expenditure is approximately doubled in mice housed singly at room temperature (22°C) vs. mice housed at thermal neutrality (30°C) (2, 17). The most significant results of this study are that (1) spontaneous activity in mice at 22°C is reduced when the mice have no access to food but increased when they are fed, (2) the energy cost of activity is doubled when the mice are housed at 22°C, (3) RMR is decreased during activity at 22°C, and (4) TEF is probably largely underestimated when measured at 22°C. Taken together, these results shed more light on how the energy expended with NST affects the components of energy expenditure and can undermine the use of mice housed below thermal neutrality as a model of human physiology.

### Spontaneous Physical Activity

We observed that activity was reduced at 22°C when the mice were fasted, but increased when they were under *ad libitum* conditions. Activity reduction in fasted mice at 22°C was primarily due to a strong decrease in the intensity of the bursts of activity and not in a reduction in the number of activity periods (Figure 3 gives a typical example of this phenomenon). It was not possible to perform such a detailed analysis of the amplitude of the bursts of activity in *ad libitum* fed mice because data acquisition was performed in a multiplexed design and measurements were performed at 10 min instead of 2 s intervals. However, we observed that the average activity intensity in mice housed at 22°C was 30% higher than in mice housed at 30°C. In addition, a percent cumulative frequency analysis of the activity data (18) indicated that fed mice housed at 22°C spent less time fully inactive and more time restless or highly active. It is probable that the low ambient temperature made the mice fell less comfortable and induced fidgeting that decreased the time spent fully inactive. On the other side of the distribution, the increased occurrence of high intensities of activity was probably related to the larger food intake induced by the increased energy requirements.

Brown and colleagues previously reported that, in fasted rats, activity was reduced at room temperature (21°C) vs. thermal neutrality (28°C) (19). They reported that video recordings indicated a cold-defensive posture at 21°C in order to decrease convective and radiant heat transfer. In our study, we did not perform video recordings but it was visually obvious that fasted mice housed at 22°C were huddled up on themselves and were rather reluctant to move. The decrease in activity in fasted mice at 22°C can therefore be the result of a behavioral adaptation to the cold to reduce heat losses in conditions where they had no opportunity to access food and refill their energy stores (20). In contrast, it seems that when under *ad libitum* feeding conditions, mice react to the decrease in room temperature by resting less, moving, and eating more. We were not able to analyze properly the food intake recordings in this study, but we observed no decrease in BW in the mice housed at 22°C during the calorimetric studies (mean δBW 22°C = 0.19 ± 0.44), which implies that mice housed at 22°C ate approximately two times more than those housed at 30°C. In contrast, under *ad libitum* conditions, Kaiyala and colleagues (21) did not report any significant effect of temperature on spontaneous physical activity. The reason for this difference remains unclear. One possible explanation may be related to the fact that, because the difference in activity under *ad libitum* conditions relies on the lowest and largest activity levels, the less precise measurement of activity with red light-beams as used by Kaiyala and colleagues, vs. force transducers used here, may indicate that they missed these differences. However, the differences in TMR at 21 and 30°C was also smaller in the Kaiyala study than in this one, and therefore, the difference may also be due to the mouse strain or sex.

### Relation between Activity, RQ, TMR, and RMR in Fasted Mice

Respiratory quotient was poorly affected by the occurrence of the bursts of activity at 22°C as well as at 30°C showing that muscle contraction was fueled by the available mixture of circulating glucose and fatty acids. Such lack of a specific increase in glucose oxidation and the strong correlation between activity and TMR probably reflects the fact that the bursts of activity were of low intensity and therefore that the work load on the muscles was low enough to be fueled by the current circulating mix of carbohydrates and lipids.

The bursts of spontaneous activity induced a rapid doubling of TMR at both 30 and 22°C. This increase reflects the energy required to fuel muscular effort. In the experiments performed to measure TEF, the high rate of data acquisition combined with the data processing by the Kalman filtering (13–15) allowed us to perform a very detailed analysis of the short-term changes between activity, TMR, and RMR in fasted mice. This analysis showed that activity marginally increased RMR at 30°C but induced curvilinear changes in RMR when the mice were housed at 22°C. At this temperature, RMR increased during the first 5 min then declined rapidly below the level measured before the onset of activity and finally reached a nadir at the end of the activity period. During the rest periods following the activity bursts, RMR returned to pre-activity values within ~30 min. The increase in RMR during the first 5 min of activity surprised us but was already described by Brown and colleagues (19), although not as precisely as here, which supports the idea that this increase was not a computational artifact. It has been suggested that the temperature set-point may be increased during activity (7) possibly to heat muscles and to improve muscular work, which may explain why this phenomenon is observed with more intensity in mice housed at 22°C than in those housed at 30°C. The following decrease in RMR reflects obviously that the heat released by the working muscles reduced the cost of thermoregulation. Accordingly, when the mice stopped moving, the heat released from the muscles progressively decreased, therefore thermal regulation was progressively restored and RMR increased back to pre-activity values. This process was also suggested by Brown and colleagues in the rat (19). According to their calculations, the decrease in what they called “supplementary thermogenesis” lasted 1–1.5 h after the end of activity. The difference may be due to the inferred timing adjustment of their equations or because the measurement were done on rats in conditions where the cold stress induced a smaller response than what we report for mice in this study (heat production was increased by only 25% instead of 100% here). On the other hand, Kaiyala and colleagues (21) reported that the thermoregulatory effort of mice housed at 21°C was reduced during the light period when activity and feeding were the highest.

The relation between the TMR increase above RMR (δMR) and the activity signal intensity computed from data acquired at a high rate showed a strong linear correlation between δMR and activity and indicated a doubling of the activity cost in fasted mice housed at 22°C. This result was confirmed in mice fed *ad libitum* where the correlation between TMR and activity, despite a less precise fit, unambiguously pointed to a significant increase of the activity cost at 22°C. Again, this phenomenon was already observed in rats by Brown and colleagues (19) who reported that the increase in heat production induced by activity was of 0.040 vs. 0.068 J/min/g^0.67^ at 28 and 21°C, respectively. Another study in which the cost of activity was investigated at different temperatures (7) also reported higher energy expended in the cold. The authors did not report directly the slope of the correlation between TMR and activity but in their discussion quoted that the energy cost per unit of activity was increased when mice were housed at low temperature (4°C). Therefore, the results of this study line up with previous reports showing that the cost of activity is increased when rats or mice are housed below thermal neutrality.

Abreu-Vieira and colleagues (7) suggested that this increase was likely due to increased heat loss from the less compact body position and disruption of the unstirred air layer around the body. However, from our data and in particular from the analysis of the very short-term changes between activity and TMR, we could observe that the extra cost of activity remained strongly correlated to the intensity of the activity signal, and therefore was produced in line with the ATP production for muscular contraction. In this context, the most plausible mechanism is an increased uncoupling between respiration and ATP production, possibly sustained by an increased expression of UCP2 and/or UCP3 in muscles to assist thermal regulation (22). It has been suggested already that variations in gene expression of UCP2 and UCP3 in muscles may affect the energy cost of exercise (23, 24). Abreu-Vieira and colleagues (7) also suggested that mice defend a higher body temperature during physical activity and that such increased uncoupling at 22°C may be a way to help increase muscle temperature. The reason for this uncoupling may be to warm the muscles and increase muscle performance at lower ambient temperatures (25). Another parameter in favor of this interpretation is, as discussed at the beginning of this section, the transient increase in RMR observed during the first 5 min of activity periods at 22°C but not at 30°C. This could be interpreted as an increased heat production by NST to warm-up the muscles at the onset of muscular effort.

### Relation between Activity, and TMR in Fed Mice

Despite the fact that, in free-feeding mice, the relation between TMR and activity measured across time in individual mice remained very strong at 22°C as well as at 30°C, and that the cost of activity at 22°C was approximately two times higher than at 30°C (as observed in fasted mice), we observed that when measured across multiple mice, the level of activity did not affect any more TMR in mice housed at 22°C and strongly weakened the relation between activity and TMR at 30°C. However, both at 22 and 30°C, AMR still strongly correlated with the amount of activity. These results are fully in line with those previously reported by Virtue et al. (8) who observed in larger groups of mice (*n* = 27) that at 30°C both total-EE and activity-EE correlated with activity while, at 24°C, only activity-EE correlated with activity. To explain the fact that the relation between TMR and activity decreases (but generally remains significant) at 30°C and is no longer observed at 22°C, one must take into account that the main determinant of TMR is RMR. In this study RMR accounted for more than 70% of TMR and *R*^2^ between TMR and RMR was above 0.90 at both temperatures. Therefore, it is not surprising that on a daily basis the activity effect on TMR be reduced by the variability in RMR, and finally vanishes below thermal neutrality where RMR fluctuates more as a result of heat transfer between thermal regulation and heat released by other discontinuous processes, such as activity and TEF. In addition, as seen when comparing Figures 2, 6 and 7, the range of TMR values available to fit the correlation with activity is much larger when measured across time in a single mouse than when measured between mice, which further weakens the correlation [see also Ref. (8)].

**Figure 10:**
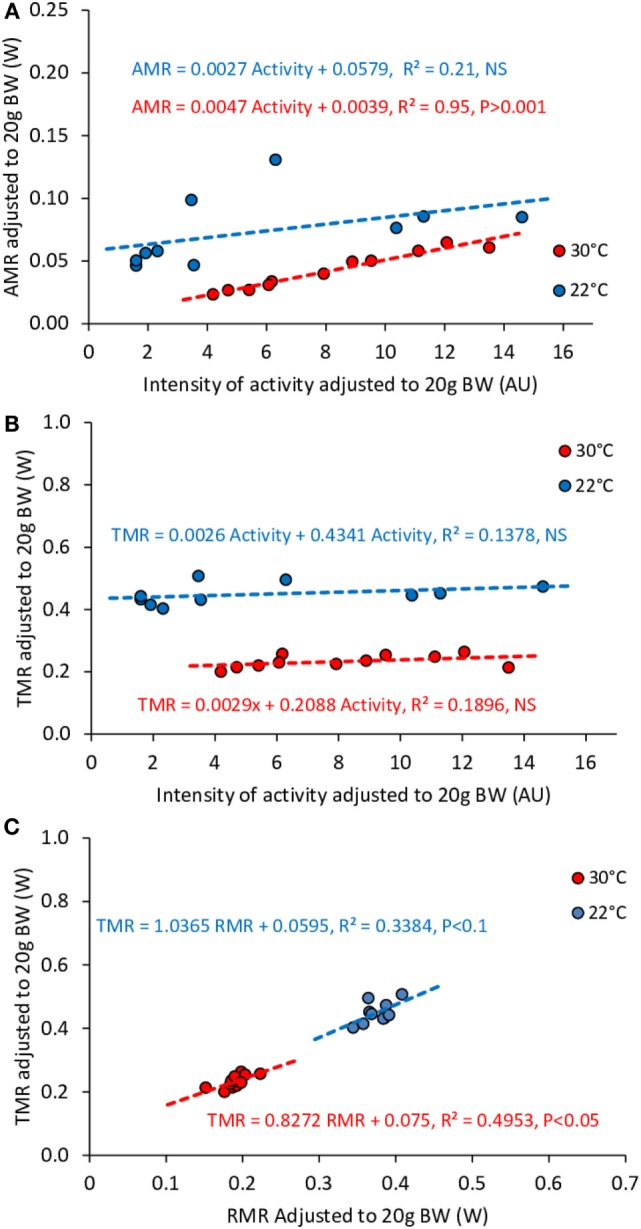
**Correlation between AMR and activity (A), TMR and activity (B), TMR and RMR (C) in fasting mice housed at 30°C (*n* = 11) and 22°C (*n* = 10)**. Data were adjusted to 20 g BW. The small ranges of activity, TMR, and RMR values in fasting mice prevented results interpretation.

Note that we performed this same analysis in fasted mice between 23:00 and 08:30 hours, i.e., when mice were in the post-absorptive state and adapted to the temperature in the metabolic cage. However, in these mice the level of activity was very low (Figure 5) and consequently individual TMR clustered around the mean group value (30°C, Mean 0.232, CV 8.93%, 22°C, mean 0.449, CV 7.48%). This prevented us from performing a precise analysis of the relation between TMR and activity across multiple mice and to reveal any effect of the activity level on TMR at 30°C as well as at 22°C (see Figure 10).

### RQ, RMR, and TEF

Resting metabolic rate in fasted mice at 22°C was two times that at 30°C, a result in line with the increase reported previously in most studies (17). In contrast, the increase in RMR induced by ingestion of the test meal at 22°C, i.e., TEF, was only one third of the response observed in mice housed at 30°C. At first glance, this could be interpreted as a strong reduction in TEF in mice housed at 22°C but, taking into account the strong interplay between heat generation for thermoregulation and heat released by muscular contraction and TEF, it is highly probable that at 22°C, fasting RMR values decreased rapidly after meal ingestion. This was confirmed by the observation that at 22°C, post-meal RMR was lower than pre-meal RMR 4 h after the meal and onward. It was not possible to measure directly the time course of the decrease in RMR after meal ingestion, but if we refer to the fast pace of changes in RMR observed in response to bursts of activity, it is possible that the cost of thermal regulation decreased within minutes after the meal was given. Therefore, at 22°C, the RMR value measured after ingestion of the test-meal was the result of the increase in RMR induced by TEF and the decrease in NST induced by the heat released from activity and TEF. In these conditions, pre-meal RMR cannot be extrapolated to compute TEF whereas this extrapolation is possible at thermal neutrality when pre-meal RMR is equal to basal metabolic rate and cannot be further decreased. An argument supporting the hypothesis that in our experimental conditions NST was much reduced by the combined effect of activity and TEF is that, when TEF was computed in reference to RMR measured 6 h after the meal, we obtained TEF values similar to those measured at 30°C. Thus, in mice at 22°C, the standard method to measure TEF cannot be applied. In conditions where it is not possible to measure precisely the decrease in NST induced by activity and TEF, it must be acknowledged that TEF cannot be accurately measured in mice housed at 22°C.

Respiratory quotient was significantly decreased after an overnight fast in mice housed at 22°C confirming that mice exhausted more quickly their glycogen stores and, after a few hours of fast had to rely on their lipid stores. The RQ response to feeding showed also that the overall increase in RQ was similar at 22 and 30°C, but that the increase was of shorter duration, reflecting the fact that mice housed at 22°C used more quickly the carbohydrates brought by the meal. Therefore not only the intensity of TEF but also the metabolic fate on the ingested nutrients is greatly affected by the increased energy demand of mice housed at 22°C.

These significant differences in TEF and RQ responses to ingestion of a test-meal at 22 and 30°C should be considered carefully because when TEF and RQ are measured in humans, great care is taken to avoid any thermal stress.

### Limitations of This Study

A main limitation in the interpretation of the results of this study is the lack of measurement of body temperature and caloric intake during calorimetry studies. The absence of caloric intake data was partly compensated for by the fact that we observed no significant changes in BW during the calorimetry studies at 22°C as well as at 30°C, indicating that energy balance was preserved and thus that caloric intake equaled total energy expenditure. In contrast, continuous online measurement of body temperature would have helped to verify that mice did not decrease their temperature set point at 22°C to reduce the cost of thermoregulation. This would have necessarily influenced the response to activity and feeding and would have provided a possible explanation for the increased cost of activity and fluctuations of RMR at 22°C. Comparison of gene expression in muscles and in white and brown adipose tissue of mice acclimatized at 22 and 30°C would have been helpful too, but in the study framework from which these data were extracted, the mice acclimatized to 30°C were reacclimatized to 22°C before organ and tissue collection.

## Conclusion

In mice housed at 22°C, resting energy expenditure is doubled by NST to maintain thermal regulation, and the cost of activity is also doubled. Intensity of NST is highest at rest and is rapidly tuned down when extra heat is released from muscular contraction and feeding. In this context, the respective roles of basal metabolic rate, NST, activity, and thermic effect of feeding in the energy balance equation are very difficult to decipher. NST in humans is most of the time close to 0. If the mouse is intended to serve as a model of human physiological regulation, it may be reasonable to house them close to thermal neutrality, in particular when they are singly housed without bedding for measurements of metabolic and behavioral parameters. On the other hand, if housing temperature is used as a tool, the mouse can be a very interesting model to study the possible role of NST in the energy balance equation.

## Author Contributions

PE and AB designed the study, performed the experiment, analyzed the data, and wrote the paper.

## Conflict of Interest Statement

The authors declare that the research was conducted in the absence of any commercial or financial relationships that could be construed as a potential conflict of interest.

## Abbreviations

AMR, activity metabolic rate; RMR, resting metabolic rate; RQ, respiratory quotient; TEF, thermic effect of feeding; TMR, total metabolic rate.
